# Potential for donation after circulatory death in Germany: a multicenter retrospective study at seven university hospitals

**DOI:** 10.3389/ti.2026.17199

**Published:** 2026-08-21

**Authors:** Jan Sönke Englbrecht, Daniel Schrader, Rainer Kram, Holger Kraus, Melanie Schäfer, Barbara Stolzenberg, Dirk Schedler, Juliane Langer, Felix Lehmann, Johannes Weller, Robert Werdehausen, Marleen Weiß, Svitlana Ziganshyna, Martin Soehle

**Affiliations:** 1 Department of Anesthesiology, Intensive Care and Pain Medicine, University Hospital Münster, Münster, Germany; 2 The Medical Director’s Staff Division of Organ Donation Coordination, University Hospital Düsseldorf, Düsseldorf, Germany; 3 Department of Anesthesiology, University Hospital Düsseldorf, Düsseldorf, Germany; 4 University Hospital Essen, Essen, Germany; 5 Department of Intensive Care Medicine, RWTH Aachen University Hospital, Aachen, Germany; 6 RWTH Aachen University Hospital, Aachen, Germany; 7 The Medical Director’s Staff Division of Organ Donation Coordination, University Hospital Cologne, Cologne, Germany; 8 University Hospital Bonn, Bonn, Germany; 9 Department of Anesthesiology and Intensive Care Medicine, University Hospital Bonn, Bonn, Germany; 10 Center for Neurology, University Hospital Bonn, Bonn, Germany; 11 Department of Anesthesiology and Intensive Care Medicine, University Medicine Magdeburg, Magdeburg, Germany; 12 University of Leipzig Medical Center, Leipzig, Germany

**Keywords:** brain death, circulatory arrest, consent, organ donation, withdrawal of life-sustaining measures

## Abstract

Many countries have established donation after circulatory death (DCD) in addition to donation after brain death (DBD). In Germany, DCD is not permitted, and its potential to increase organ donation rates has not yet been quantified. We retrospectively identified all deceased with brain injury between 2023–2024 at seven German university hospitals. Eligible DCD donors were defined as patients with preserved brainstem reflexes precluding DBD donation, documented consent or an unknown donation preference, and death occurring within 120 min after withdrawal of life-sustaining measures. For patients with unknown preference, a consent rate of 40% was assumed. Among 4,587 deceased, 1,509 had preserved brainstem reflexes as the main reason against DBD. Of these, 58 patients with documented consent and 212 patients with an unknown donation preference died within 120 min after treatment withdrawal. Compared with 128 utilized DBD donors, this corresponds to an additional theoretical DCD potential of 45% based solely on documented consent. When applying the assumed consent rate to patients with an unknown preference, the hypothetical increase reaches 112%. The introduction of a DCD program could substantially increase organ donation rates at German university hospitals. However, its actual impact would depend on establishing appropriate legal, organizational, and ethical frameworks.

## Graphical Abstract

**Figure:**
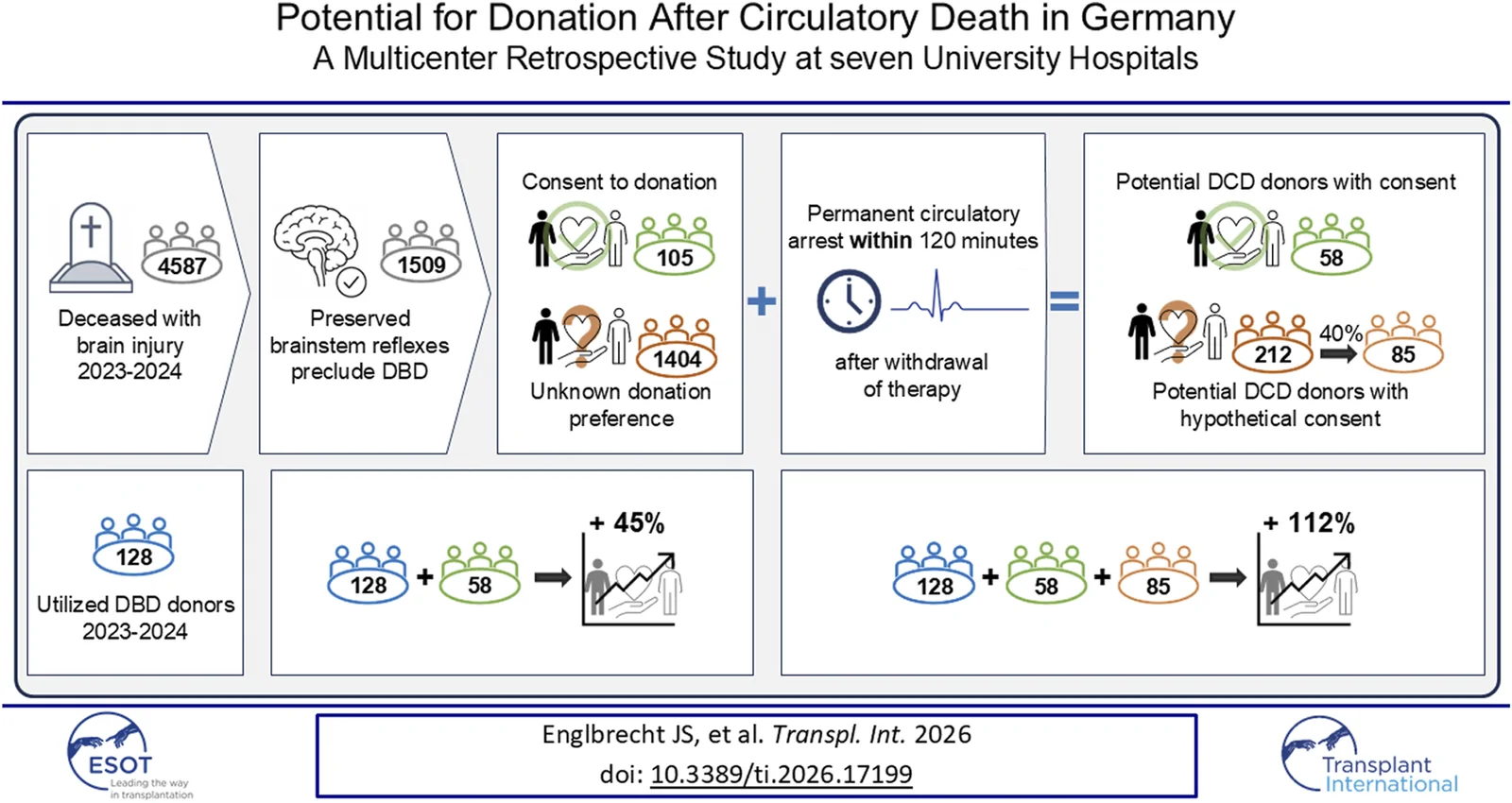

## Introduction

Given the persistent disparity between organ supply and demand, many countries have complemented donation after brain death (DBD), based on the determination of brain death/death by neurologic criteria (BD/DNC), with programs for donation after circulatory death (DCD) [1, 2].

DCD donors are commonly classified according to the Maastricht criteria into four (five in countries formally recognizing the possibility of organ donation after euthanasia) categories [3, 4]. Among these, category III DCD donors account for the largest proportion of DCD procedures (controlled donation after circulatory determination of death (cDCDD)) [1]. These donors are typically patients with a devastating brain injury who do not meet the criteria for BD/DNC, but whose prognosis is considered so poor that withdrawal of life-sustaining measures (WLSM) is deemed appropriate in the patient’s best interests [5]. Death subsequently occurs following permanent circulatory arrest after WLSM. The interval between WLSM and circulatory arrest is referred to as the agonal phase [4]. Determination of death after WLSM is based on the occurrence of permanent circulatory arrest. Permanence—and thus death—is confirmed after a predefined observation period to exclude autoresuscitation. This so-called “No-touch” period between circulatory arrest and formal death determination typically ranges from 5 to 20 min, depending on national regulations [1, 6, 7].

Organ quality in DCD donation is significantly influenced by the duration of warm ischemia. Total warm ischemia time (WIT) encompasses both the agonal phase and the interval between circulatory arrest and the start of cold perfusion, or, when used, normothermic regional perfusion [6]. Functional warm ischemic time (FWIT) represents a shorter period and is generally defined as the interval from the onset of sustained organ hypoperfusion following WLSM—typically marked by a decline in blood pressure and/or oxygen saturation—to the initiation of *in situ* preservation [6]. To minimize ischemic injury and preserve graft viability, many transplant programs apply maximum acceptable FWIT thresholds ranging from 30 to 120 min, depending on the organ to be transplanted [2, 7, 8].

In 2024, Germany had the lowest deceased donor rate within the Eurotransplant region, with 10.9 donors per million population [9]. The lack of a DCD program may contribute to this persistently low donation rate [10, 11]. Evidence from countries with established DCD programs indicates that DCD can substantially increase the donor pool without adversely affecting DBD activity [12–16]. Nevertheless, German regulations currently do not recognize permanent circulatory arrest as equivalent to death determined by BD/DNC. Consequently, DCD donation and the transplantation of organs recovered from DCD donors are not permitted in Germany [17]. In this context, some German-language publications use outdated terms such as “cardiac arrest” or “non-heart-beating donation.” In relation to cDCDD, these terms refer to permanent circulatory arrest following planned WLSM rather than an unexpected cardiac arrest.

To date, estimates of the potential contribution of DCD to organ donation in Germany have relied largely on modelling studies and extrapolations from international experience, while empirical data on potential DCD donors are lacking [16]. Therefore, this study sought to quantify the number of potential cDCDD donors among deceased patients with severe brain injury at seven German university hospitals.

## Patients and methods

This study was performed in accordance with the Declaration of Helsinki. The Ethics Committee of the University of Muenster approved the study protocol (file number 2021-801-f-S) on 28 May 2025. The need for informed consent was waived due to the retrospective analysis of routinely collected patient data.

All patients with primary and/or secondary brain injury who died during hospitalization between January 2023 and December 2024 at seven German university hospitals (Aachen, Bonn, Düsseldorf, Essen, Leipzig, Magdeburg, and Münster) were retrospectively included. These patients were identified using *TransplantCheck*, a screening tool provided by the German Organ Procurement Organization (DSO). The software analyzes routinely collected hospital discharge data submitted in accordance with Section 21 of the German Hospital Remuneration Act (*Krankenhausentgeltgesetz*) and identifies all deceased patients with primary and/or secondary brain injury [18].

Cases with absolute contraindications to organ donation, patients not receiving mechanical ventilation, and all utilized DBD donors were excluded. The remaining cases underwent a case-by-case medical record review to identify patients in whom preserved brainstem reflexes (BSR), precluding the determination of BD/DNC, represented the principal reason for not proceeding to DBD donation. Patients with a previously documented objection to organ donation or whose medical condition precluded organ donation (e.g., suspected malignancy or severe organ dysfunction) were excluded from further analysis.

Subsequently, the medical records were reviewed to assess whether the patient’s organ donation preferences had been evaluated. Cases with preserved BSR and documented consent to organ donation were classified as potential DCD donors with consent (pDCD-C), whereas cases without documented evaluation were classified as potential DCD donors with unknown donation preference (pDCD-U).

Assessment of the agonal phase followed a stepwise retrospective procedure for all pDCD-C and pDCD-U. First, medical records were reviewed to determine whether complete WLSM had been performed at a clearly identifiable time point and whether death subsequently occurred within 120 min. Complete WLSM was defined as withdrawal of all relevant life-sustaining measures, including discontinuation of hemodynamic support and mechanical ventilation. Because sustained organ hypoperfusion following WLSM is a key predictor of transplant outcomes, a predefined eligibility window of 120 min after complete WLSM was applied based on established cDCDD practice [2, 7, 19]. Once a case did not meet these eligibility criteria (complete WLSM could not be clearly identified or death did not occur within 120 min), no further time-to-death data were abstracted. Consequently, these two reasons could not be quantified separately among cases not meeting the eligibility criteria. The exact duration of the agonal phase was recorded only for patients who died within 120 min after complete WLSM. These cases were subsequently classified as eligible DCD donors with consent (eDCD-C) and eligible DCD donors with unknown donation preferences (eDCD-U), respectively.

For all eDCD-C and eDCD-U, the following data were collected:

- Age and sex
- Type of brain injury
- Length of intensive care unit stay
- Performance of decompressive neurosurgical surgery
- Notification to the DSO
- Documented organ donation preference

To estimate the number of cases within the eDCD-U cohort that might have consented to organ donation if donation preferences had been evaluated, published consent rates for DBD donation were used. Reported consent rates for DBD donation in Germany range from 37% to 53% [9, 10, 20]. Therefore, a conservative hypothetical consent rate of 40% was applied to the eDCD-U cohort [21]. The number of eligible DCD donors with hypothetical consent (eDCD-hC) was estimated as 40% of the eDCD-U cohort (eDCD-hC = eDCD-U x 0.4).

The total number of eligible DCD donors was calculated as the sum of eDCD-C and eDCD-hC. The theoretical increase in organ donation activity was determined relative to the number of DBD donors utilized during the study period.

The study-specific donor categories used in this analysis were derived from the Critical Pathway for Deceased Donation terminology [22] and adapted for a retrospective assessment of DCD potential in the absence of a DCD program. Consequently, the categories “potential DCD donor” and “eligible DCD donor” were defined according to predefined study criteria and do not correspond directly to the respective donor categories used within the terminology.

### Statistical methods

Statistical analyses were performed using SPSS Statistics (IBM Corp., Version 31). Categorical variables are presented as absolute and relative frequencies and were compared using the Pearson chi-square test. Fisher’s exact test was applied when expected cell counts were less than five. For dichotomous variables, odds ratios (OR) with 95% confidence intervals (CI) were calculated as measures of effect size. Continuous variables were assessed for normality using the Shapiro–Wilk test. As most variables were not normally distributed, they are presented as median and interquartile range (IQR; 25th–75th percentile). Group comparisons were performed using the Mann–Whitney U test. All statistical tests were two-sided, and a p-value <0.05 was considered statistically significant.

## Results

### Eligible DCD donors

**FIGURE 1:**
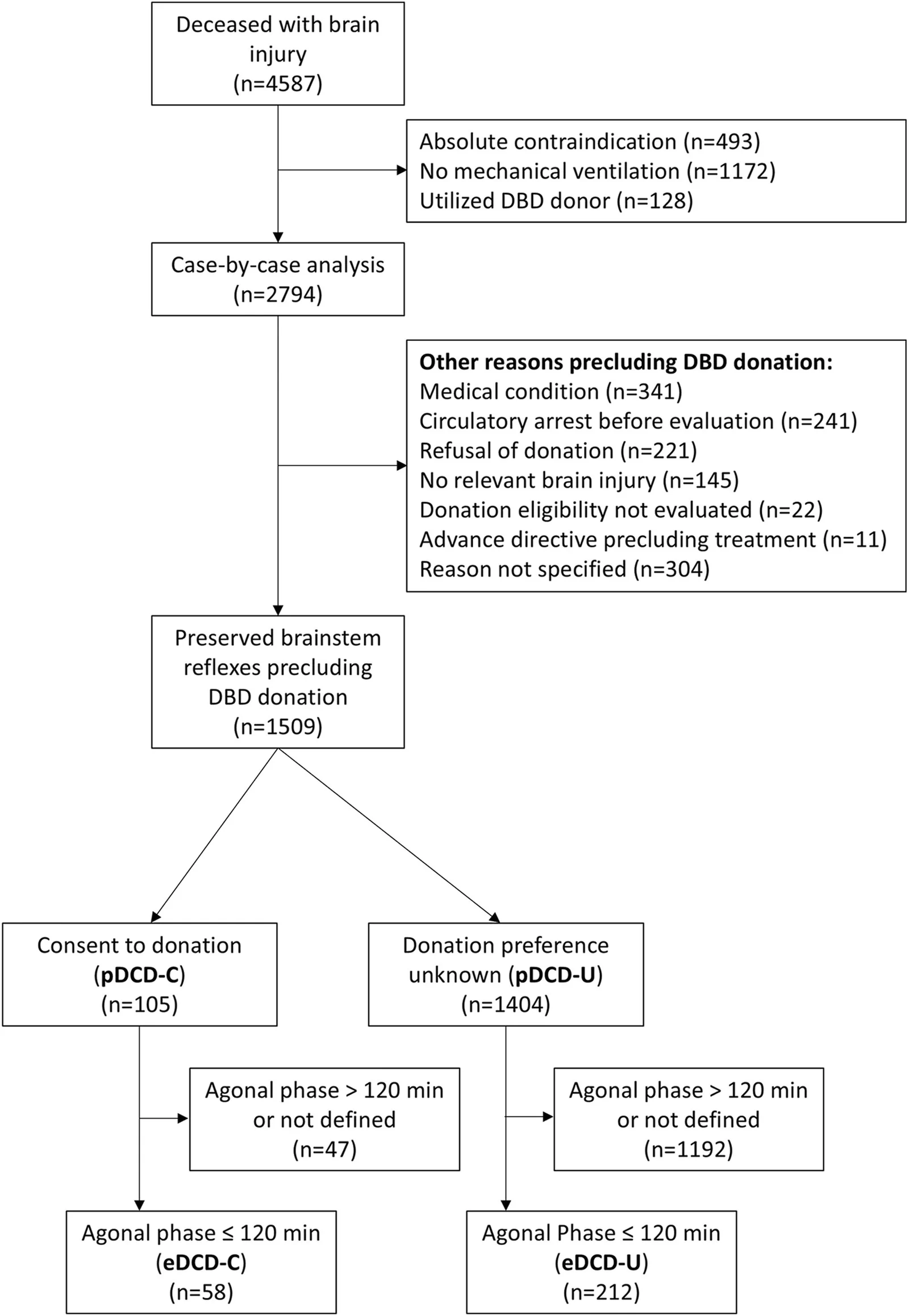
Flow chart of the study cohort. DBD: donation after brain death, pDCD-C, potential donation after circulatory death donor with consent; pDCD-U, potential donation after circulatory death donor with unknown donation preference; eDCD-C, eligible donation after circulatory death donor with consent; eDCD-U, eligible donation after circulatory death donor with unknown donation preference.

A total of 4,587 deceased patients with documented brain injury were identified. After exclusion of utilized DBD donors, patients with absolute contraindications to donation and patients without mechanical ventilation, 2,794 patients underwent a case-by-case analysis. Among these, preserved BSR represented the primary reason for ineligibility for DBD donation in 1,509 cases (54%). The remaining 1,285 cases (46%) had other primary reasons for ineligibility for DBD donation. These included medical condition precluding donation (27%), circulatory arrest before evaluation (19%), refusal of organ donation (17%), absence of relevant brain injury (11%), lack of evaluation of donation eligibility (2%), and an advance directive precluding further treatment (1%). In 24% of cases, the reason was not further specified in the medical records. Among the 1,509 patients with preserved BSR, documented consent to organ donation was available in 105 cases (7%; pDCD-C), whereas organ donation preferences had not been evaluated in 1,404 cases (93%; pDCD-U) (Figure 1).

Overall, 270 of the 1,509 patients with preserved BSR (18%) had a clearly identifiable time point of complete WLSM and died within 120 min thereafter. The remaining 1,239 patients were not assessed further once either complete WLSM could not be clearly identified or death did not occur within 120 min. The proportion of patients with an agonal phase within 120 min was significantly higher among pDCD-C than among pDCD-U, resulting in 58 eDCD-C and 212 eDCD-U, respectively (55.2% vs. 15.1%; OR 6.94, 95% CI 4.60–10.47; p < 0.001). Overall, the median agonal phase among the 270 eligible DCD donors was 29 min [IQR 15–50; range 0–117]. In the eDCD-C cohort, the median agonal phase was 19 min [IQR 13–35; range 0–117]. Thirty-nine patients (67%) died within 30 min, 13 (22%) between 31 and 60 min, and 6 (10%) between 61 and 120 min. In the eDCD-U cohort, the median agonal phase was 32 min [IQR 17–53; range 0–117], with 101 patients (48%) dying within 30 min, 66 (31%) between 31 and 60 min, and 45 (21%) between 61 and 120 min.

Compared with eDCD-U, eDCD-C had a significantly shorter agonal phase, were younger, and were more frequently reported to the DSO. Intracerebral hemorrhage was more common and ischemic stroke less common among eDCD-C. No significant difference was observed regarding the performance of decompressive neurosurgical surgery. Among eDCD-C cases, consent was most frequently based on presumed will communicated by a substitute decision maker (Table 1).

**TABLE 1:** Eligible donation after circulatory death donors with agonal phase ≤120 min.

Variable	eDCD-C (n = 58)	eDCD-U (n = 212)	p-value
Agonal phase (minutes)	19 [13–35]	32 [17–53]	0.002
Age (years)	59 [49–74]	72 [63–81]	<0.001
Sex • Male • Female	37 (63.8%) 21 (36.2%)	124 (58.5%) 88 (41.5%)	0.466
Type of brain injury • Intracranial hemorrhage • Hypoxic brain injury • Ischemic stroke • Traumatic brain injury • Other	30 (51.7%) 15 (25.9%) 7 (12.1%) 4 (6.9%) 2 (3.4%)	66 (31.1%) 58 (27.4%) 60 (28.3%) 18 (8.5%) 10 (4.7%)	0.029
Intensive care unit length of stay (hours)	126 [76–214]	137 [66–263]	0.560
Neurosurgical decompressive surgery • Yes • No	14 (24.1%) 44 (75.9%)	37 (17.5%) 175 (82.5%)	0.249
Notification to the DSO • Yes • No	33 (56.9%) 25 (43.1%)	6 (2.8%) 206 (97.2%)	<0.001
Type of consent • Written declaration • Verbal declaration • Presumed will (as stated by SDM) • SDM own values • Not assessed	14 (24.1%) 14 (24.1%) 27 (46.6%) 3 (5.2%) 0	- - - - 212	​

Categorical variables are presented as absolute and relative frequencies, and continuous variables as median [IQR]. eDCD-C, eligible donation after circulatory death donor with consent, eDCD-U, eligible donation after circulatory death donor with unknown donation preference, DSO, German organ procurement organization, SDM, substitute decision maker.

### Theoretical increase in organ donation activity through DCD

Based on 128 utilized DBD donors during the study period, inclusion of all eDCD-C would theoretically increase the donor pool by 45%. Applying a conservative hypothetical consent rate of 40% to the eDCD-U cohort yielded an additional 85 eDCD-hC. The combined inclusion of eDCD-C and eDCD-hC would result in a theoretical overall increase in donor numbers of 112% compared with utilized DBD donations alone (Table 2).

**TABLE 2:** Number of organ donors by cohort.

Cohort	n (%)
Case-by-case analysis • eDCD with consent (eDCD-C) • eDCD with unknown donation preference (eDCD-U) ○ With hypothetical consent (eDCD-hC = eDCD-U x 0.4) • With consent (eDCD-C) or hypothetical consent (eDCD-hC)	2,794 (100%) 58 (2.1%) 212 (7.6%) 85 (3.0%) 143 (5.1%)
Potential increase • Utilized DBD donors (reference) • DBD + eDCD-C • DBD + eDCD-C + eDCD-hC	128 186 (+45%) 271 (+112%)

Categorical variables are presented as absolute and relative frequencies. eDCD-C, eligible donation after circulatory death donor with consent, eDCD-U, eligible donation after circulatory death donor with unknown donation preference, eDCD-hC, eligible donation after circulatory death donor with hypothetical consent.

## Discussion

Our data indicate a considerable untapped potential for DCD donation in German university hospitals. Based solely on eligible DCD donors with documented consent, donor numbers at the participating centers could theoretically have increased by up to 45%. Applying a conservative hypothetical consent rate of 40% to eligible DCD donors with unknown donation preferences yielded a projected overall 112% increase compared with utilized DBD donations alone.

### Potential of eligible DCD donors

At first glance, the proportion of eDCD-C among all cases undergoing case-by-case chart review (2.1%) appears relatively small. However, this cohort represents only cases in which consent to organ donation had been evaluated. This does not imply that organ donation would have been declined in all remaining cases. Rather, it reflects the current regulatory framework governing organ donation in Germany. In patients with preserved BSR, organ donation preferences may often remain unevaluated because a diagnosis of BD/DNC cannot be established and DBD donation is therefore not feasible [18]. Similarly, although refusal of organ donation was documented as the primary reason against DBD donation in only 8% of patients undergoing case-by-case analysis, this should not be interpreted as a representative refusal rate, because donation preferences were often not evaluated when other medical or procedural reasons had already precluded DBD donation.

The limited availability of documented consent also raises broader questions regarding the current German opt-in system. A transition to an opt-out system would change how the absence of a documented donation preference is handled and might increase the number of patients in whom donation can be considered. However, longitudinal evidence indicates that changing the consent default alone does not necessarily increase donation rates. Effective donor identification and referral, public awareness, communication with relatives, and sustained public acceptance remain essential [11, 23–25]. Accordingly, the introduction of a DCD program may expand the donor pool but is unlikely to resolve organ scarcity as a standalone measure and should form part of a broader strategy to strengthen organ donation.

Nevertheless, because donation preferences had been evaluated in only a small proportion of potentially eligible cases, the eDCD-C cohort likely underestimated the true DCD potential. To address this limitation, a hypothetical consent rate was applied as previously used in comparable studies [21]. Using this approach, eligible DCD donors with documented or hypothetical consent accounted for 5.1% of all cases undergoing individual chart review. Despite differences in methodology and donor definitions, this proportion is broadly consistent with findings from international studies, which have identified potential DCD donors in 2.8%–8.6% of patients dying in intensive care units [21, 26–28].

However, the application of a hypothetical consent rate has further limitations. Available data on consent rates for DBD donation in Germany are heterogeneous. While representative surveys indicate a generally favorable attitude towards organ donation, with approximately 73% of individuals who have made a decision expressing support for donation [29], consent rates in the actual donation process appear substantially lower. A major contributing factor is the frequent absence of documented donation preferences, leaving the substitute decision maker to decide on behalf of the patient, often resulting in refusal of donation [10, 30].

An additional limitation is that the hypothetical consent rate applied in the present analysis was derived from data on DBD donation and may not be directly transferable to the DCD setting. Existing evidence regarding consent rates for DCD donation is inconsistent. One study reported lower consent rates for DCD than for DBD donation (55% vs. 64%), whereas another found higher overall donation consent rates in countries where both DBD and DCD programs were available [31, 32]. Consequently, it remains uncertain whether, and to what extent, the introduction of a DCD program would influence consent rates in Germany. The estimates presented in this study should therefore be interpreted as hypothetical projections rather than precise predictions of future donation activity.

Another important limitation is that the available data does not allow determination of how many of the identified eligible DCD donors would ultimately have met the medical criteria for organ donation. Such an assessment would only be possible during a real donor evaluation process. Although cases with absolute contraindications to organ donation were excluded *a priori* and the age of eDCD-C was comparable to that of utilized DBD donors in Germany [9], additional factors may have precluded donation. Analyses of established cDCDD programs demonstrate that donor pathways are progressively reduced by medical contraindications, DCD-specific eligibility criteria, lack of authorization, death occurring outside the predefined time window following WLSM, and final organ-specific suitability [5, 27]. In a large US cohort, approximately two-thirds of authorized and medically suitable potential DCD donors ultimately proceeded to donation, although this proportion depends on program characteristics and the definitions applied [33]. Data from the UK national DCD program indicate that adult DCD donors yield, on average, 2.9 transplantable organs per donor [34]. Therefore, although each cDCDD donor may provide multiple transplantable organs and implementation of cDCDD would likely result in a substantial increase in transplant activity, estimating the exact number of additional transplantations arising from our retrospective cohort would require assumptions beyond the scope of this study. Accordingly, the estimated increases of 45% and 112% were not adjusted for subsequent non-utilization within a real cDCDD pathway and should therefore be interpreted as theoretical donor potential rather than expected increases in realized donation activity.

Moreover, our analysis cannot determine whether this additional donor potential would translate into a proportional increase in transplant activity or measurable improvements in waiting-list outcomes. International data indicate that utilization is lower for DCD than for DBD donors and varies considerably by organ type [35]. Kidneys represent the most established DCD grafts, whereas utilization of pancreata remains more restricted [36, 37]. There is also evidence that post-transplant outcomes may differ between DCD and DBD grafts depending on the organ and clinical context [36, 38]. These aspects could not be assessed in the present study and can only be evaluated prospectively following implementation of a German DCD program, including organ-specific utilization, transplantation activity, waiting-list outcomes, and graft- and patient-centered outcomes.

Another limitation is that only deceased with a diagnosed brain injury were included in the analysis. This approach may underestimate the overall potential of DCD donation, as DCD is also feasible in patients with other underlying conditions. However, experience from countries with established DCD programs indicates that donors without brain injury account for only a small proportion of utilized DCD donations [13, 39].

The study cohort comprised 128 utilized DBD donors across the participating centers during the two-year study period, representing less than 10% of all utilized DBD donations in Germany. Nevertheless, the donor conversion rate (utilized DBD donors divided by all deceased patients with brain injury) was 2.8%, exceeding the national average across all donor hospitals in Germany (1.5%) and closely matching that reported for German university hospitals overall (2.7%) [18, 40]. These findings suggest that the observed relative increase in donor numbers is unlikely to be substantially inflated by unusually low DBD donation rates at the participating centers.

Because only university hospitals were included, the findings cannot be directly extrapolated to all donor hospitals. In Germany, the 38 university hospitals account for approximately 35% of all utilized DBD donors, while hospitals with neurosurgical services (133 centers) contribute a further 42%, and hospitals without neurosurgical services (948 centers) account for the remaining donors [9]. University hospitals therefore achieve the highest number of utilized DBD donors per institution. However, patient populations, case mix, and disease severity differ considerably between hospital types and levels of care and are likely to influence the number of potential DCD donors. Consequently, the DCD potential identified in this study cannot be assumed to apply to hospitals of other levels of care. Moreover, the participating centers represented only three of Germany’s 16 federal states and therefore constituted a regional rather than a nationally representative sample. Regional differences in case mix, referral patterns and end-of-life practices may also influence DCD potential.

### Agonal phase

The type and extent of treatment limitations applied to patients with a poor prognosis are not standardized and likely vary considerably between hospitals [41]. Overall, both withholding and withdrawal of life-sustaining therapies in German intensive care units have become increasingly common [42, 43]. For a DCD program to function effectively, clear criteria for prognostic assessment, particularly regarding the anticipated timing of death after WLSM, as well as standardized end-of-life care protocols are required [5, 44].

Two prospective multicenter studies reported that circulatory arrest occurred within 1 hour after WLSM in 55%–76% of potential DCD donors and within 2 hours in 63%–83% [45, 46]. However, no patient-related factors have yet been consistently identified that reliably predict the duration of the agonal phase [2, 45]. Consequently, accurate prediction of death following WLSM remains one of the major challenges in the implementation of cDCDD programs.

As Germany has no established DCD program, no standardized DCD-specific WLSM protocol was in place in our cohort. The observed agonal phase therefore reflects routine end-of-life care rather than a structured DCD pathway and should be interpreted with caution regarding its transferability to a future cDCDD program. In addition, the exact agonal phase was recorded only for patients who died within the predefined 120-min eligibility window, precluding assessment of the complete time-to-death distribution. The overall proportion of patients with preserved BSR dying within 120 min was 18%, substantially lower than reported in prospective DCD cohorts. However, these populations are not directly comparable, as previous studies included preselected potential DCD donors undergoing planned WLSM, whereas our denominator comprised all retrospectively identified patients with preserved BSR. Notably, 55.2% of the pDCD-C cohort died within 120 min, compared with 15.1% of pDCD-U. The lower overall proportion was therefore largely driven by the large pDCD-U cohort. Nevertheless, retrospective case identification and the absence of a standardized DCD-specific WLSM process may have introduced selection or misclassification bias.

Despite these limitations, a clear difference between the cohorts remained evident. The odds of an agonal phase within 120 min were nearly sevenfold higher in the pDCD-C cohort than in the pDCD-U cohort. Furthermore, the eDCD-C cohort exhibited a significantly shorter agonal phase and was more frequently reported to the DSO than the eDCD-U cohort. These findings suggest that organ donation preferences were more likely to be evaluated in patients with more severe brain injury and a higher likelihood of progressing to BD/DNC. The greater severity of neurological injury may also explain the shorter time to death following WLSM observed in this group. Conversely, less severe brain injury may have been associated with a lower expectation of progression to BD/DNC and, consequently, a lower likelihood of organ donation evaluation. This interpretation is supported by the higher prevalence of intracerebral hemorrhage among eDCD-C and by recent German data demonstrating that assessment of organ donation preferences is performed substantially less frequently when progression to BD/DNC is not anticipated than when it is expected [47].

Some authors have argued that DCD should be considered in all potential DCD donors [45]. While prognostic tools such as the Wisconsin criteria and the DCD-N score have been developed to estimate the likelihood of circulatory arrest following WLSM, reliable and generalizable patient-related predictors of death within a predefined time frame after WLSM remain lacking [44, 48, 49]. As proposed by others, our findings indicate that clinical judgement by intensive care physicians may play an important role in identifying patients likely to experience a short agonal phase [48]. This observation is particularly relevant if organ donation discussions within a DCD program are to be limited to situations in which DCD donation represents a realistic possibility, thereby avoiding unnecessary burden on patients’ families and caregivers.

### Ethical implications

Our findings highlight that a substantial proportion of patients in Germany with a devastating brain injury and documented consent to organ donation are currently unable to donate because they do not fulfil the legal and medical requirements for organ donation. Consequently, opportunities for organ transplantation may be lost despite the patient’s clearly documented wish to donate. Our findings therefore reveal more than an untapped donor potential. They demonstrate a discrepancy between patients’ documented preferences and the options currently provided within the German legal framework. This discrepancy has recently been addressed in a German interdisciplinary analysis of medical, ethical, and legal perspectives on DCD [50]. The analysis highlights the situation of patients who may be medically suitable donors but remain excluded from donation because they do not fulfil the criteria for BD/DNC. It further emphasizes that a documented willingness to donate represents an important expression of personal autonomy, while such consent does not establish an individual entitlement to organ donation and must be balanced against the requirements of appropriate end-of-life care and reliable determination of death. Any future introduction of a DCD program would therefore require strict independence of WLSM decisions, proportionate organ-preserving measures, and safeguards maintaining public trust. These ethical considerations should form an integral part of any future societal, political, and legislative debate on the introduction of cDCDD in Germany.

### Limitations

Several limitations should be considered when interpreting the findings of this study. First, the classification of patients into eDCD-C and eDCD-U was not based on predefined inclusion criteria but rather reflected the existing clinical documentation. Consequently, comparisons between these groups should be interpreted with caution, as systematic differences between them cannot be excluded.

Second, the agonal phase rather than the clinically more relevant FWIT was used for donor eligibility assessment. This approach was necessary because the available data did not allow determination of FWIT. Furthermore, because no standardized DCD-specific WLSM protocol was in place and exact time to death was recorded only within the predefined 120-min window, the observed agonal phase distribution may not be fully representative of a future cDCDD program.

As discussed above, the most important limitations of the potential analysis are the low proportion of deceased patients in whom organ donation preferences had been evaluated, the exclusion of patients without underlying brain injury, and the restriction of the study population to university hospitals. Together, these factors may have influenced the estimated number of potential and eligible DCD donors and limit the generalizability of the findings.

## Conclusion

This multicenter retrospective study suggests that the introduction of cDCDD could substantially increase organ donation activity at German university hospitals. Even when considering only eligible DCD donors with documented consent, donor numbers could theoretically increase by up to 45%. The ultimate impact of a DCD program, however, would depend not only on donor availability but also on the establishment of appropriate legal, organizational, and ethical frameworks.

Implementation of a DCD program would require standardized approaches to WLSM, reliable identification of potential DCD donors, and broad ethical and societal consensus. Given Germany’s low donation rate within the Eurotransplant region, the establishment of a successful DCD program has been advocated as a matter of particular importance not only for Germany but also for the wider Eurotransplant community [51]. The findings of the present study support this view by demonstrating a substantial untapped DCD donor potential in German university hospitals.

## Data Availability

The data analyzed in this study is subject to the following licenses/restrictions: The datasets generated and analysed during the current study are not publicly available due to the inclusion of sensitive patient data. Anonymized raw data supporting the conclusions of this article will be made available on reasonable request. Requests to access these datasets should be directed to jan.englbrecht@ukmuenster.de.

## References

[1] Lomero M Gardiner D Coll E Haase‐Kromwijk B Procaccio F Immer F Donation after circulatory death today: an updated overview of the European landscape. Transpl Int (2020) 33:76–88. 10.1111/tri.13506 31482628

[2] Smith M Dominguez-Gil B Greer DM Manara AR Souter MJ . Organ donation after circulatory death: current status and future potential. Intensive Care Med (2019) 45:310–21. 10.1007/s00134-019-05533-0 30725134

[3] Kootstra G Daemen JH Oomen AP . Categories of non-heart-beating donors. Transpl Proc (1995) 27:2893–4. 7482956

[4] Thuong M Ruiz A Evrard P Kuiper M Boffa C Akhtar MZ New classification of donation after circulatory death donors definitions and terminology. Transpl Int (2016) 29:749–59. 10.1111/tri.12776 26991858

[5] Domínguez-Gil B Ascher N Capron AM Gardiner D Manara AR Bernat JL Expanding controlled donation after the circulatory determination of death: statement from an international collaborative. Intensive Care Med (2021) 47:265–81. 10.1007/s00134-020-06341-7 33635355 PMC7907666

[6] Martin DE Cillo U Berman M Glazier AK Miñambres E Opdam H Seeking global agreement on controlled donation after circulatory determination of death: methodology and definitions of the Bucharest international European Society for Organ Transplantation (ESOT) consensus. Transpl Int (2026) 39:16280. 10.3389/ti.2026.16280 42294092 PMC13254459

[7] Krendl FJ Oberparleiter S Ponholzer F Messner F Scheidl S Maglione M Implementation of a controlled DCD program in Western Austria – key considerations and insights. Transpl Int (2026) 39:16147. 10.3389/ti.2026.16147 42367327 PMC13306392

[8] Kalisvaart M Croome KP Hernandez-Alejandro R Pirenne J Cortés-Cerisuelo M Miñambres E Donor warm ischemia time in DCD Liver transplantation-working group report from the ILTS DCD, liver preservation, and machine perfusion consensus conference. Transplantation (2021) 105:1156–64. 10.1097/TP.0000000000003819 34048418

[9] Deutsche Stiftung Organtransplantation. Jahresbericht Organspende und Transplantation in Deutschland 2025 [Annual Report on Organ Donation and Transplantation in Germany 2025] (2026). Available online at: https://dso.de/SiteCollectionDocuments/DSO-Jahresbericht_2025.pdf (Accessed April 23, 2026).

[10] Englbrecht JS Schrader D Kraus H Schäfer M Schedler D Bach F How large is the potential of brain dead donors and what prevents utilization? A multicenter retrospective analysis at seven university hospitals in North Rhine-Westphalia. Transpl Int (2023) 36:11186. 10.3389/ti.2023.11186 37252613 PMC10211426

[11] Tackmann E Dettmer S . Measures influencing post-mortem organ donation rates in Germany, the Netherlands, Spain and the UK: a systematic review. Anaesthesist (2019) 68:377–83. 10.1007/s00101-019-0600-4 31101922

[12] Summers DM Counter C Johnson RJ Murphy PG Neuberger JM Bradley JA . Is the increase in DCD organ donors in the United Kingdom contributing to a decline in DBD donors? Transplantation (2010) 90:1506–10. 10.1097/TP.0b013e3182007b33 21079550

[13] Hodgson R Young AL Attia MA Lodge JPA . Impact of a national controlled donation after circulatory death (DCD) program on organ donation in the United Kingdom: a 10-Year study. Am J Transpl (2017) 17:3172–82. 10.1111/ajt.14374 28556608

[14] Weiss J Elmer A Béchir M Brunner C Eckert P Endermann S Deceased organ donation activity and efficiency in Switzerland between 2008 and 2017: achievements and future challenges. BMC Health Serv Res (2018) 18:876. 10.1186/s12913-018-3691-8 30458762 PMC6247533

[15] Gäbel M Gyllström Krekula L Lindblom H Karud K Hildebrand K Löwhagen Hendén P 306.7: controlled DCD in Sweden – national implementation, expansion and future directions. Transplantation (2025) 109:S63. 10.1097/01.tp.0001175096.63318.79

[16] von Samson-Himmelstjerna FA de Ferrante H Niehus CB Strelniece A Kakavand N Thomsen S-Y Cardiac arrest as an extended indication for organ donation: an analysis of potential in Germany based on retrospective data from European countries. Dtsch Arztebl Int (2026) 123:207–12. 10.3238/arztebl.m2026.0004 41674356 PMC13274318

[17] Englbrecht JS Holling M . Organ donation after determination of brain death: legal aspects and practical approach. Anaesthesiologie (2023) 72:67–78. 10.1007/s00101-022-01241-5 36637499 PMC9839225

[18] Brauer M Günther A Pleul K Götze M Wachsmuth C Meinig T How many potential organ donors are there really? retrospective analysis of why determination of irreversible loss of brain function was not performed in deceased patients with relevant brain damage. Anaesthesist (2019) 68:22–9. 10.1007/s00101-018-0510-x 30446808

[19] Scalea JR Redfield RR Arpali E Leverson GE Bennett RJ Anderson ME Does DCD donor time-to-death affect recipient outcomes? Implications of time-to-death at a high-volume center in the United States. Am J Transpl (2017) 17:191–200. 10.1111/ajt.13948 27375072

[20] Wesslau C Grosse K Krüger R Kücük O Mauer D Nitschke F-P How large is the organ donor potential in Germany? Results of an analysis of data collected on deceased with primary and secondary brain damage in intensive care unit from 2002 to 2005. Transpl Int (2007) 20:147–55. 10.1111/j.1432-2277.2006.00413.x 17239023

[21] Rakhra SS Opdam HI Gladkis L Arcia B Fink MA Kanellis J Untapped potential in Australian hospitals for organ donation after circulatory death. Med J Aust (2017) 207:294–301. 10.5694/mja16.01405 28954604

[22] Domínguez-Gil B Delmonico FL Shaheen FAM Matesanz R O’Connor K Minina M The critical pathway for deceased donation: reportable uniformity in the approach to deceased donation. Transpl Int (2011) 24:373–8. 10.1111/j.1432-2277.2011.01243.x 21392129

[23] Dallacker M Appelius L Brandmaier AM Morais AS Hertwig R . Opt-out defaults do not increase organ donation rates. Public Health (2024) 236:436–40. 10.1016/j.puhe.2024.08.009 39305662

[24] Abbasi M Najafizadeh K Latifi M Ghobadi O Sadeghi M-H Zali A . Impact of opt-in *versus* opt-out organ donation legislation on donation rates: a systematic review. J Perioper Pract (2026):17504589251390742. 10.1177/17504589251390742 41618506

[25] Arshad A Anderson B Sharif A . Comparison of organ donation and transplantation rates between opt-out and opt-in systems. Kidney Int (2019) 95:1453–60. 10.1016/j.kint.2019.01.036 31010718

[26] Dias FS Fernandes DM Cardoso-Fernandes A Silva A Basílio C Gatta N Potential for organ donation after controlled circulatory death: a retrospective analysis. Porto Biomed J (2024) 9:259. 10.1097/j.pbj.0000000000000259 38993948 PMC11236395

[27] Mone T Rosenthal T Seto T . Direct measurement of DCD donor potential. Transplantation (2025) 109:715–9. 10.1097/TP.0000000000005188 39233321 PMC11927442

[28] Tenza E Valero R Arraez V . Estimation of potential donors after cardiocirculatory death in elche university general hospital (Alicante, Spain). Med Intensiva (2017) 41:153–61. 10.1016/j.medin.2016.08.003 27979629

[29] Zimmering R Hammes D . Bericht zur Repräsentativstudie 2022 “Wissen, Einstellung und Verhalten der Allgemeinbevölkerung zur Organ-und Gewebespende”. In: Bzga Forschungsbericht. [Report on the 2022 Representative Study “Knowledge, Attitudes and Behavior of the General Population Towards Organ and Tissue Donation in Germany”]. Köln: Bundeszentrale Für Gesundheitliche Aufklärung (BZgA) (2023). Available online at: https://www.bioeg.de/fileadmin/user_upload/PDF/studien/BZGA-23-05444-Repraesentativbefragung-Organspende-2022.pdf (Accessed July 23, 2026).

[30] Englbrecht JS Schrader D Kraus H Schäfer M Schedler D Bach F Advance directives and consent to organ donation in seven university hospitals in north Rhine-Westphalia-a retrospective, multicenter analysis. Dtsch Arztebl Int (2023) 120:133–4. 10.3238/arztebl.m2022.0367 37083573 PMC10154790

[31] Prescott J Gardiner D Hogg L Harvey D . How the mode of organ donation affects family behaviour at the time of organ donation. J Intensive Care Soc (2019) 20:204–7. 10.1177/1751143718807842 31447912 PMC6693122

[32] Qu Z Oedingen C Bartling T Schrem H Krauth C . Factors influencing deceased organ donation rates in OECD countries: a panel data analysis. BMJ Open (2024) 14:e077765. 10.1136/bmjopen-2023-077765 PMC10882290 38387981

[33] Nelson HM Glazier AK Delmonico FL . Changing patterns of organ donation: brain dead donors are not being lost by donation after circulatory death. Transplantation (2016) 100:446–50. 10.1097/TP.0000000000000954 26516669

[34] NHS Blood and Transplant. Organ and tissue donation and transplantation activity report 2024/2025 (2025). Available online at: https://www.organdonation.nhs.uk/about-organ-donation/statistics-about-organ-donation/transplant-activity-report/ (Accessed July 20, 2026).

[35] Martin F Carmona M Mahillo B Alvarez M Luengo A Chatzixiros E Organ donation and transplantation worldwide: the global observatory on donation and transplantation 2024 report. Transplantation (2026) 110:e655–e669. 10.1097/TP.0000000000005657 41670431 PMC12908642

[36] Paessler A Brierley J Siebelink M Loukopoulos I Kessaris N Stojanovic J . Long-term outcomes of pediatric kidney transplants from DCD and DBD donors: a comparative OPTN study. Transpl Int (2025) 38:14706. 10.3389/ti.2025.14706 41140298 PMC12548547

[37] Patel C Kourounis G van Leeuwen L Holzner M Wadhera V Akhtar MZ Modelling donor factors influencing pancreas transplant utilization and evolution of decision-making over time. Commun Med (2026) 6:231. 10.1038/s43856-026-01506-9 41795015 PMC13096168

[38] Frasco PE Mathur AK Chang Y-H Alvord JM Poterack KA Khurmi N Days alive and out of hospital after liver transplant: comparing a patient-centered outcome between recipients of grafts from donation after circulatory and brain deaths. Am J Transpl (2023) 23:55–63. 10.1016/j.ajt.2022.10.007 36695622

[39] Joint Faculty of Intensive Care Medicine of Ireland. Donation after Circulatory Death. The Intensive Care Society of Ireland. Joint Faculty of Intensive Care Medicine of Ireland Website (2016). Available online at: https://jficmi.anaesthesia.ie/wp-content/uploads/2016/12/ICSI-Guideline-DCD.pdf (Accessed May 27, 2026).

[40] Englbrecht JS Schrader D Alders JB Schäfer M Soehle M . Post-COVID-19 pandemic organ donation activities in Germany: a multicenter retrospective analysis. Front Public Health (2024) 12:1356285. 10.3389/fpubh.2024.1356285 38444435 PMC10912160

[41] Kotsopoulos AMM Böing-Messing F Jansen NE Vos P Abdo WF . External validation of prediction models for time to death in potential donors after circulatory death. Am J Transpl (2018) 18:890–6. 10.1111/ajt.14529 28980398

[42] Denke C Jaschinski U Riessen R Bercker S Spies C Ragaller M End-of-life practices in 11 German intensive care units. Med Klin Intensivmed Notfmed (2023) 118:663–73. 10.1007/s00063-022-00961-1 36169693 PMC10624715

[43] Sprung CL Ricou B Hartog CS Maia P Mentzelopoulos SD Weiss M Changes in end-of-life practices in European Intensive care units from 1999 to 2016. JAMA (2019) 322:1692–704. 10.1001/jama.2019.14608 31577037 PMC6777263

[44] Opdam H Pérez-Blanco A Gardiner D Hasz R Jansen NEE Le Dorze M Establishing standards for controlled donation after circulatory determination of death in adults: the Bucharest international European Society for Organ transplantation consensus. Transpl Int (2026) 39:16284. 10.3389/ti.2026.16284 42326389 PMC13276612

[45] Wind J Snoeijs MGJ Brugman CA Vervelde J Zwaveling J van Mook WN Prediction of time of death after withdrawal of life-sustaining treatment in potential donors after cardiac death*. Crit Care Med (2012) 40:766–9. 10.1097/CCM.0b013e318232e2e7 21983365

[46] Kotsopoulos A Vos P Witjes M Volbeda M Franke H Epker J Prospective multicenter observational cohort study on time to death in potential controlled donation after circulatory death donors-development and external validation of prediction models: the DCD III study. Transplantation (2022) 106:1844–51. 10.1097/TP.0000000000004106 35266926

[47] Lehmann F Ehrentraut SF Görtzen-Patin J Söhle M Langer J Banat M HEW score-a tool for the homogenisation of donor registrations to the DSO: Multicentre retrospective analysis of three university hospitals. Med Klin Intensivmed Notfmed (2025) 120:653–660. 10.1007/s00063-024-01237-6 39873738 PMC12594715

[48] Lewis J Peltier J Nelson H Snyder W Schneider K Steinberger D Development of the university of Wisconsin donation after cardiac death evaluation tool. Prog Transpl (2003) 13:265–73. 10.1177/152692480301300405 14765718

[49] Rabinstein AA Yee AH Mandrekar J Fugate JE de Groot YJ Kompanje EJO Prediction of potential for organ donation after cardiac death in patients in neurocritical state: a prospective observational study. Lancet Neurol (2012) 11:414–9. 10.1016/S1474-4422(12)70060-1 22494955

[50] Simon A Duttge G , editors. Donation After Cardiac Death (DCD): Eine Landkarte Medizinischer, Ethischer Und Rechtlicher Perspektiven [Donation After Cardiac Death (DCD): A Map of Medical, Ethical, and Legal Perspectives]. Göttingen: Göttingen University Press (2026). 10.17875/gup2026-3085

[51] Eden J Dutkowski P Schlegel A . Reply to: “how many liver grafts could be recovered after implementation of donation after cardiac death in Germany?”. J Hepatol (2023) 79:e120–e121. 10.1016/j.jhep.2023.05.014 37245704

